# A multi‐view learning approach with diffusion model to synthesize FDG PET from MRI T1WI for diagnosis of Alzheimer's disease

**DOI:** 10.1002/alz.14421

**Published:** 2024-12-06

**Authors:** Ke Chen, Ying Weng, Yueqin Huang, Yiming Zhang, Tom Dening, Akram A. Hosseini, Weizhong Xiao

**Affiliations:** ^1^ School of Computer Science University of Nottingham Ningbo China Ningbo China; ^2^ School of Computer Science University of Nottingham Nottingham UK; ^3^ Institute of Mental Health NHC Key Laboratory of Mental Health (Peking University) National Clinical Research Center for Mental Disorders (Peking University Sixth Hospital) Beijing China; ^4^ School of Medicine University of Nottingham Nottingham UK; ^5^ Neurology Department Nottingham University Hospitals NHS Trust Queen's Medical Centre Nottingham UK; ^6^ Neurology Department Peking University Third Hospitals Beijing China

**Keywords:** Alzheimer's disease, data synthesis, diffusion model, fluorodeoxyglucose positron emission tomography, magnetic resonance imaging T1 weighted imaging, multi‐view learning

## Abstract

**INTRODUCTION:**

This study presents a novel multi‐view learning approach for machine learning (ML)–based Alzheimer's disease (AD) diagnosis.

**METHODS:**

A diffusion model is proposed to synthesize the fluorodeoxyglucose positron emission tomography (FDG PET) view from the magnetic resonance imaging T1 weighted imaging (MRI T1WI) view and incorporate two synthesis strategies: one‐way synthesis and two‐way synthesis. To assess the utility of the synthesized views, we use multilayer perceptron (MLP)–based classifiers with various combinations of the views.

**RESULTS:**

The two‐way synthesis achieves state‐of‐the‐art performance with a structural similarity index measure (SSIM) at 0.9380 and a peak‐signal‐to‐noise ratio (PSNR) at 26.47. The one‐way synthesis achieves an SSIM at 0.9282 and a PSNR at 23.83. Both synthesized FDG PET views have shown their effectiveness in improving diagnostic accuracy.

**DISCUSSION:**

This work supports the notion that ML‐based cross‐domain data synthesis can be a useful approach to improve AD diagnosis by providing additional synthesized disease‐related views for multi‐view learning.

**Highlights:**

- We propose a diffusion model with two strategies to synthesize fluorodeoxyglucose positron emission tomography (FDG PET) from magnetic resonance imaging T1 weighted imaging (MRI T1WI).
- We raise multi‐view learning with MRl T1Wl and synthesized FDG PET for Alzheimer's disease (AD) diagnosis.
- We provide a comprehensive experimental comparison for the synthesized FDG PET view.
- The feasibility of synthesized FDG PET view in AD diagnosis is validated with various experiments.
- We demonstrate the ability of synthesized FDG PET to enhance the performance of machine learning–based AD diagnosis.

## 1 BACKGROUND

In practical scenarios, a multitude of information manifests in diverse formats as heterogeneous data sources describe an entity. In the medical field, data acquisition frequently encompasses a variety of domains or modalities. For instance, magnetic resonance imaging (MRI) and positron emission tomography (PET) can be categorically conceptualized as multi‐view data.^1^

Multi‐view learning can exploit complementary information of multiple views of data or information to improve the performance of the machine learning (ML) model.^2^, ^3^, ^4^ The medical data in the artificial intelligence (AI) domain is limited, making it challenging to generalize. By exploiting multi‐view learning, different medical data modalities can be treated as a view, and many researchers have found that it can improve model performance and generalization.^5^ In most medical applications, using multi‐modality medical images can produce better results than using only one modality medical image, because different modalities of medical images reflect different pathologies and also provide different views for specific diseases.^6^, ^7^

The World Alzheimer Report 2018^8^ estimated that the number of people suffering from dementia was ≈ 50 million in 2018 and will more than triple to 152 million by 2050. Alzheimer's disease (AD) is the most common cause of dementia, accounting for 60% to 80% of dementia cases, and is one of the main causes of physical and mental health disorders especially among older people.^9^ Preliminary cognitive tests using multiple scales, such as the Mini‐Mental State Examination (MMSE)^10^ and Montreal Cognitive Assessment (MoCA),^11^ are most commonly used for AD diagnosis. However, these cognitive tests are affected by education level, language, and so forth, which implies they are insufficiently objective as a means of diagnosis. The neuroimaging examination is a more objective and precise test that can be used for the diagnosis or early diagnosis of AD as well as to distinguish it from other types of dementia.

Structural MRI (sMRI) is the most common modality for AD diagnosis due to its low cost of acquisition, non‐invasiveness, and accessibility. Various ML methods using sMRI have been proposed for early diagnosis and prediction of AD as well as for exploring brain structural pathological patterns.^12^, ^13^, ^14^, ^15^, ^16^, ^17^, ^18^, ^19^, ^20^ PET is a relatively new modality of neuroimaging with fewer data existing due to its high expense and limited access to scanners compared to the sMRI, though the usage of PET is steadily increasing worldwide. Recent studies have shown the effectiveness of using PET with the assistance of ML techniques for the early diagnosis and prediction of AD.^12^, ^20^, ^21^, ^22^ Multi‐view learning approaches can also assist in AD diagnosis and prognosis.^21^, ^23^

The multi‐view learning of sMRI and PET has also been recognized as a useful method for the early diagnosis and prediction of AD.^24^, ^25^, ^26^ The combination of sMRI and PET scans can provide both structural and functional information related to AD, therefore enabling ML methods to enhance the effectiveness and robustness of the diagnosis.

The motivation behind this study lies in addressing the limitations posed by the availability and cost of PET scans, which are valuable yet often inaccessible for many patients. Given the high cost and limited accessibility of PET imaging, there is an imminent need to develop alternative methods that can provide similar diagnostic benefits. We explore the possibility of synthesizing fluorodeoxyglucose PET (FDG PET) images from sMRI data, which is more widely available and less costly. FDG PET can reflect neuronal activity by measuring cerebral glucose metabolism and hence can offer valuable insights into metabolic changes, even beyond obvious anatomical brain changes such as brain atrophy, which makes it a powerful tool for early diagnosis and monitoring of AD progression. The early diagnosis of AD is crucial for identifying the risk of AD progression and for earlier intervention. Furthermore, the use of FDG PET combined with other imaging modalities, such as sMRI, can provide a comprehensive view of both functional and structural changes in the brain, hence enriching the understanding of disease mechanisms and progression. By leveraging multi‐view learning and advanced image synthesis techniques, we aim to enhance the accuracy and accessibility of AD diagnosis. Recent studies^27^, ^28^, ^29^, ^30^, ^31^, ^32^, ^33^ have also shown the feasibility of synthesizing FDG PET scans from sMRI scans with various ML‐based methods.

**FIGURE 1:**
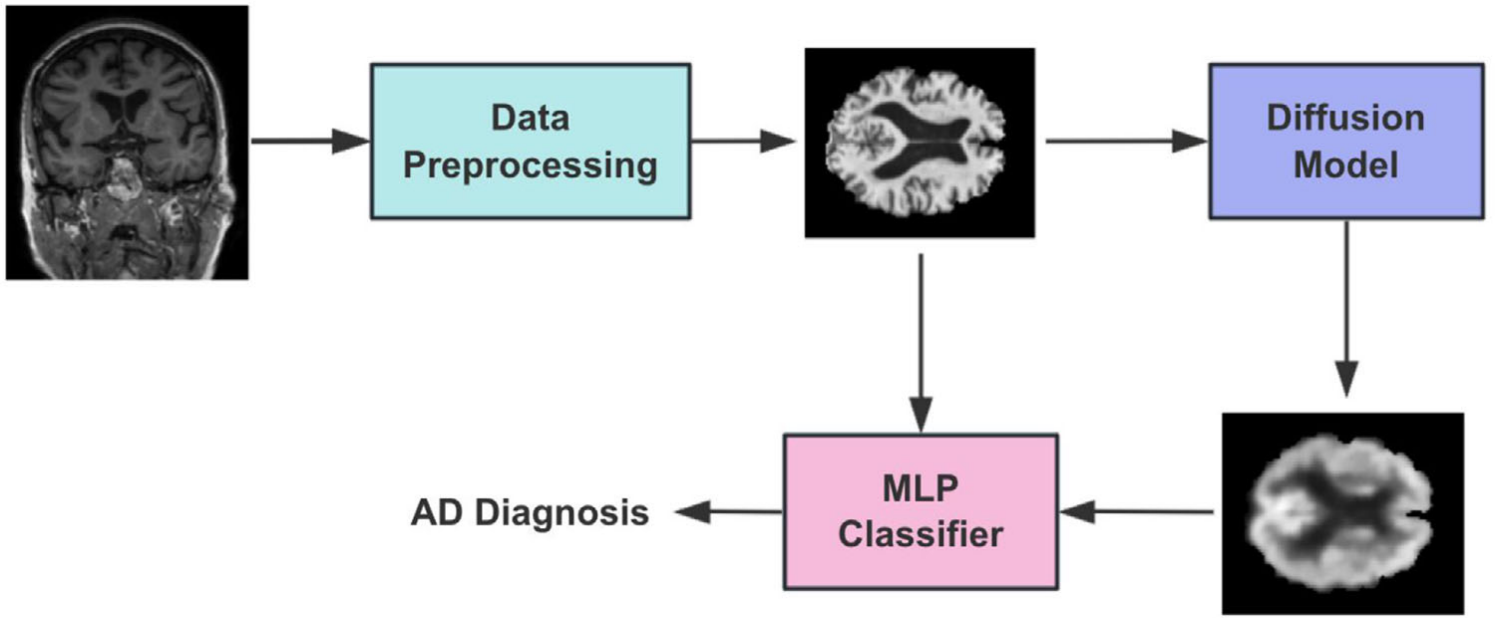
Pipeline of the framework. First, raw MRI T1WI scans are preprocessed to derive standardized preprocessed scans. Then the preprocessed MRI T1WI scans are fed into the diffusion model to synthesize the FDG PET. Furthermore, both preprocessed MRI T1WI scans and synthesized FDG PET scans are jointly fed into the classifier to provide an AD diagnosis. AD, Alzheimer's disease; FDG PET, fluorodeoxyglucose positron emission tomography; MLP, multilayer perceptron; MRI T1WI, magnetic resonance imaging T1 weighted imaging.

This study is clinically significant as it proposes a novel approach that could improve the early detection and diagnosis of AD. By synthesizing FDG PET images from sMRI data, specifically MRI T1 weighted imaging (MRI T1WI) scans, we provide a cost‐effective, accessible, and non‐invasive method to enhance diagnostic accuracy. This approach is beneficial for settings with limited access to FDG PET imaging, and will ultimately make contributions to better patient outcomes by enabling earlier and more accurate diagnosis. The overall pipeline is shown in Figure 1. The raw sMRI scans are first preprocessed to derive the corresponding skull removed and standard space registered scans. Then the preprocessed scans are fed into the diffusion model to generate the synthesized PET scans. Last, the preprocessed scans and the synthesized PET scans are jointly fed into the classifier to make a prediction of AD progression stage.

## 2 METHOD

### 2.1 Datasets

Data used in the preparation of this work were obtained from the Alzheimer's Disease Neuroimaging Initiative (ADNI) database (adni.loni.usc.edu). ADNI was launched in 2003 as a public–private partnership, led by Principal Investigator Michael W. Weiner, MD. The primary goal of ADNI is to test whether serial MRI, PET, other biological markers, and clinical and neuropsychological assessment can be combined to measure the progression of mild cognitive impairment (MCI) and early AD.

In this research, we have downloaded the paired MRI T1WI and FDG PET scans for the task of synthesizing FDG PET scans from MRI T1WI scans. The pairing procedure is based on the metadata of the downloaded images and the terms for pairing are the same subject ID and the interval of the date of obtaining the image < 30 days. The demographic analysis of the dataset with MRI PET pairs is shown in Table 1.

**TABLE 1 alz14421-tbl-0001:** Demographic analysis of paired dataset with MRI T1WI scans and FDG PET scans as pairs.

Age	76.59 ± 6.65
Sex	
Male	208
Female	124
Subject	332
Scan	1035

Abbreviations: FDG PET, fluorodeoxyglucose positron emission tomography; MRI T1WI, magnetic resonance imaging T1 weighted imaging.

**RESEARCH IN CONTEXT**

1. **Systematic review**: Literature on machine learning (ML)–based Alzheimer's disease (AD) diagnosis and data synthesis in the medical field has been investigated. Many studies support the effectiveness of using neuroimaging for AD diagnosis as well as involving multi‐view data. Some studies also show the potential to use synthesized data in the medical field.
2. **Interpretation**: We propose a novel multi‐view learning approach for the diagnosis of AD using ML, which involves data synthesis through a diffusion model. Our proposed two‐way diffusion model generates high‐quality synthesized data, and extensive experiments show the effectiveness of this synthesized data in ML–based AD diagnosis.
3. **Future directions**: The results of our study demonstrate the potential of using synthesized data to augment the performance of computer‐assisted diagnosis (CAD). Expanding the synthesis of diverse data modalities, particularly those that are costly yet valuable, would greatly benefit CAD. Further advancements in high‐quality data synthesis methods are imperative for enhancing the diagnostic precision of CAD.

### 2.2 Data preprocessing

The data in the ADNI dataset were collected from different sources, which introduces some differences existing in voxel size, orientation, and so forth. To make the data representation consistent, data preprocessing was carried out using the FMRIB Software Library (FSL).^34^ For both MRI T1WI scans and FDG PET scans, we removed non‐brain tissue at the first step to reduce the bias in the following data preprocessing steps. The images were then registered to the standard Montreal Neurological Institute 152 (MNI152) space to help further data preprocessing and experiments. Moreover, to extract more refined feature representation for AD diagnosis, brain region segmentation was applied to the registered images to obtain white matter (WM), gray matter (GM), and cerebrospinal fluid (CSF).

### 2.3 Diffusion model

**FIGURE 2:**
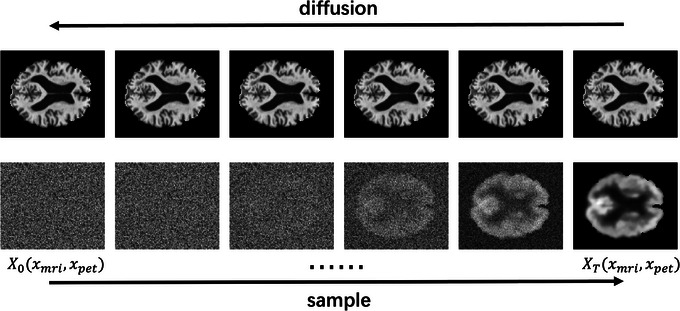
Illustration for diffusion and sample process.

Diffusion models, also known as denoising diffusion probabilistic models (DDPMs),^35^ are latent[Table alz14421-tbl-0001] variable models that define a diffusion process and a reversed diffusion process, also known as the sample process. In the diffusion process, Gaussian noise is continuously added to the input data to obtain its latent structure in the Gaussian distribution space while the noisy data are gradually denoised to obtain the expected output in the input data distribution space in the sample process. Figure 2 shows an example of diffusion progress in the second row including a diffusion process from right to left that diffuses the input data by gradually adding noise and a sample process from left to right that denoises the noisy input to reverse generate the desired noise‐free image. The goal of a diffusion model is to learn the way of modeling the data into the latent Gaussian distribution space, equivalently the diffusion process.

In this research, the aim is to generate FDG PET scans from MRI T1WI scans, which implies that the MRI T1WI scans should be the input and the FDG PET scans should be the denoised output image. However, instead of directly learning the way to map the input to the latent Gaussian distribution space for both modalities of images with a uniform model, we use the MRI T1WI scans as condition images that are used to guide the diffusion and denoising progress as shown in Figure 2, where the first channel of the input is always MRI T1WI and the noising/denoising is only applied on the second channel. Specifically, MRI T1WI scans are used in an additional channel apart from their corresponding FDG PET scan in the diffusion process to train the model for the learning of space mapping from FDG PET scans to Gaussian noises. In the sampling process, MRI T1WI scans are also used in an additional channel apart from noisy data to guide the denoising steps to recover their corresponding FDG PET scans.

With the FDG PET channel denoted as $x_{\mathrm{PET}}$ and the MRI T1WI channel as $x_{MRI}$, the input image is denoted as X, which contains two channels $\left(x_{\mathrm{PET}},x_{\mathrm{MRI}}\right)$. The general idea is gradually adding a small amount of noise to the input data to generate a series of noisy data $\left\{X_{0},X_{1},\text{…}X_{T}\right\}$ for many time steps T. However, in our design, the noise is only added on the $x_{\mathrm{PET}}$ at each time step while the $x_{MRI}$ remains the same. The noise level of *X* is then steadily increased from 0 to T. A U‐NET Diff is used to predict the noise used for generating *X_t_*_–1_ from *X_t_*. During the training, with the ground truth for $X_{0}$ and a Gaussian noise *ϵ*, an *X_t_* can be derived as following:

(1) $$X_{t}=\sqrt{{\bar{\alpha }}_{t}}\;X_{0}+\sqrt{1-{\bar{\alpha }}_{t}}\varepsilon$$

where $\alpha_{t}=1-\beta_{t}$ and ${\bar{\alpha }}_{t}=\Pi_{s=1}^{t}\;\alpha_{s}$. $\beta_{1}$ to $\beta_{t}$ is a set of predefined variances.

The U‐NET model then takes the *X_t_* as input to predict its corresponding noise and is trained using an MSE loss with *ϵ*.

(2) $$\mathrm{MSE}\;\left(Diff\right)=\mathrm{mean}{\left(\varepsilon -Diff\left(\sqrt{{\bar{\alpha }}_{t}}X_{0}+\sqrt{1-{\bar{\alpha }}_{t}}\varepsilon \right)\right)}^{2}$$

Meanwhile, because the parts of the image other than brain tissue are not used for the FDG PET synthesis, we restrict the loss only to the brain tissue by applying a brain mask *Mask_brain_* as follows:

(3) $$\mathrm{Loss}\;\left(Diff\right)=\mathrm{mean}{\left(Mask_{brain}\left(\varepsilon -Diff\left(\sqrt{{\bar{\alpha }}_{t}}X_{0}+\sqrt{1-{\bar{\alpha }}_{t}}\varepsilon \right)\right)\right)}^{2}$$

During the sample process used for generating the FDG PET, according to the formulation for DDPMs, *X_t_*_–1_ can be derived from *X_t_* as follows:

(4) $$\;x_{t-1}=\sqrt{{\bar{\alpha }}_{t-1}}\;\left(\frac{x_{t}-\sqrt{1-{\bar{\alpha }}_{t}}Diff\left(X_{t}\right)}{\sqrt{{\bar{\alpha }}_{t}}}\right)+\sqrt{1-{\bar{\alpha }}_{t-1}-\sigma_{t}^{2}}Diff\left(X_{t}\right)+\sigma_{t}\varepsilon$$

where $\sigma_{t}=\sqrt{\frac{1-{\bar{\alpha }}_{t-1}}{1-{\bar{\alpha }}_{t}}}\;\sqrt{1-\frac{{\bar{\alpha }}_{t}}{{\bar{\alpha }}_{t-1}}}$, which is used to control the degree of adding stochastic noise at each step. Unlike a normal image generating task that requires diversity in the generated images, it is expected to be deterministic in FDG PET synthesized from MRI T1WI. Therefore, we set the $\sigma_{t}$ to 0 as described in denoising diffusion implicit models (DDIMs)^36^ to produce a deterministic sampling. Meanwhile, this enables speeding up sample progress by rescaling the total time steps T to a smaller value.

**FIGURE 3:**
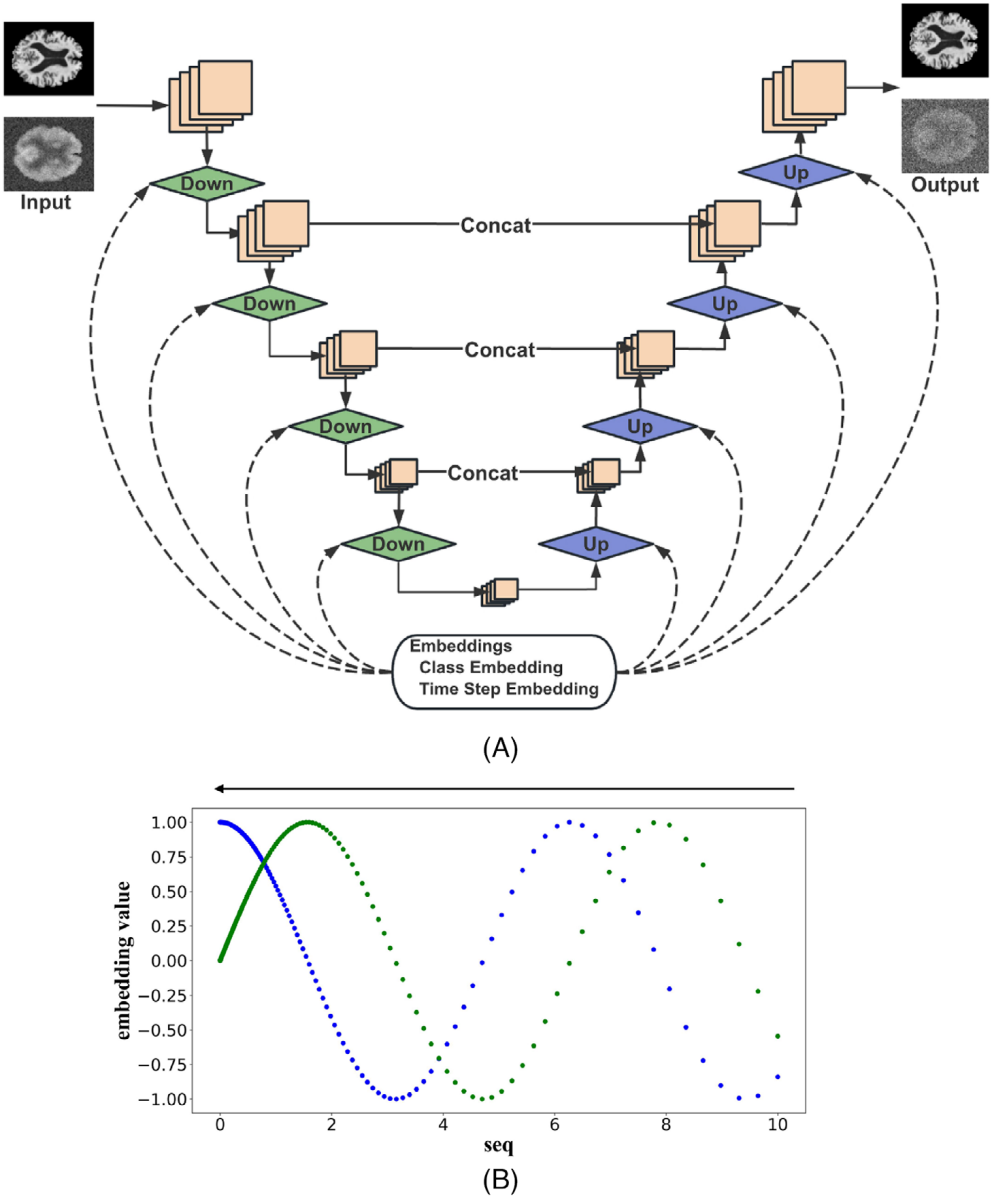
A, Structure of the diffusion model. B, An example of time step embedding at the 10th time step. The blue points denote the first 256 elements of the time step embedding related to the sin function and the green points denote the remaining 256 elements of the time step embedding related to the cos function. The y axis denotes the embedding value and the x axis denotes the seq variable in Equation (5). The order of the elements arranged in the time step embedding is from right to left as the arrow shows.

The backbone of the diffusion model is also a U‐NET–style model with several steps of down‐sampling modules to extract high‐level features and several steps of up‐sampling modules to reconstruct the high‐resolution output. A skip connection is also included to enhance the model's output by merging lower level but higher resolution features into the expanding path, equivalent to the continuous up‐sampling process. Moreover, special embeddings are added to the network as additional inputs, namely class embedding and time step embedding. In our work, time step embedding is a cos‐sin embedding defined as following:

(5) $$\mathrm{seq}=\left[e^{-\log\left(\mathrm{mp}\right)\times i}\right]\;\times \mathrm{timestep}$$

(6) $${\mathrm{emb}}_{\mathrm{timestep}}=\mathrm{concat}\left(\left[\sin\left(\mathrm{seq}\right),\cos\left(\mathrm{seq}\right)\right]\right)$$

where mp denotes for maximum period which is set to 10,000, i ranges from 0 to 255 in this work, and concat denotes the concatenation operation. To summarize, the time step embedding in this work has a dimension of 512 where the first 256 elements are related to the sin function and the remaining are related to the cos function. An example visualization of time step embedding is shown in Figure 3B with blue referring to the first 256 elements and green referring to the remaining 256 elements. The order of the elements is from right to left.

After generating the sin‐cos time step embedding based on a specific time step, the time step embedding would further be fed into fully connected networks consisting of linear layers and non‐linear activation layers to deeply embed the time step:

(7) $$\begin{array}{c} emb_{\mathrm{timestep}}^{\mathrm{deep}}=fc\left(\mathrm{em}b_{\mathrm{timestep}}\right) \end{array}$$

where fc denotes the fully connected networks for deeply embedding the time step.

Class embedding is used for identifying which modality of data is going to be sampled or diffused. In this study, the data to be used could be either MRI T1WI or FDG PET scans. The class embedding is defined as a set of learnable parameters which has the same dimension as the time step embedding. In other words, both of the classes, MRI T1WI and FDG PET, have their corresponding learnable class embedding variable with a length of 512.

The final embedding fed into Down and Up modules as shown in Figure 3A is a combination of time step embedding and class embedding as follows:

(8) $$\begin{array}{c} emb_{\mathrm{timestep}}^{\mathrm{deep}}=emb_{\mathrm{timestep}}^{\mathrm{deep}}+emb_{\mathrm{class}}\; \end{array}$$

Therefore, the final embedding allows the model to receive information on which time step it is and refines the prediction with the assistance of time step embedding. Moreover, the final embedding also determines the model's output direction because it contains class embedding, which is an output prompt.

**FIGURE 4:**
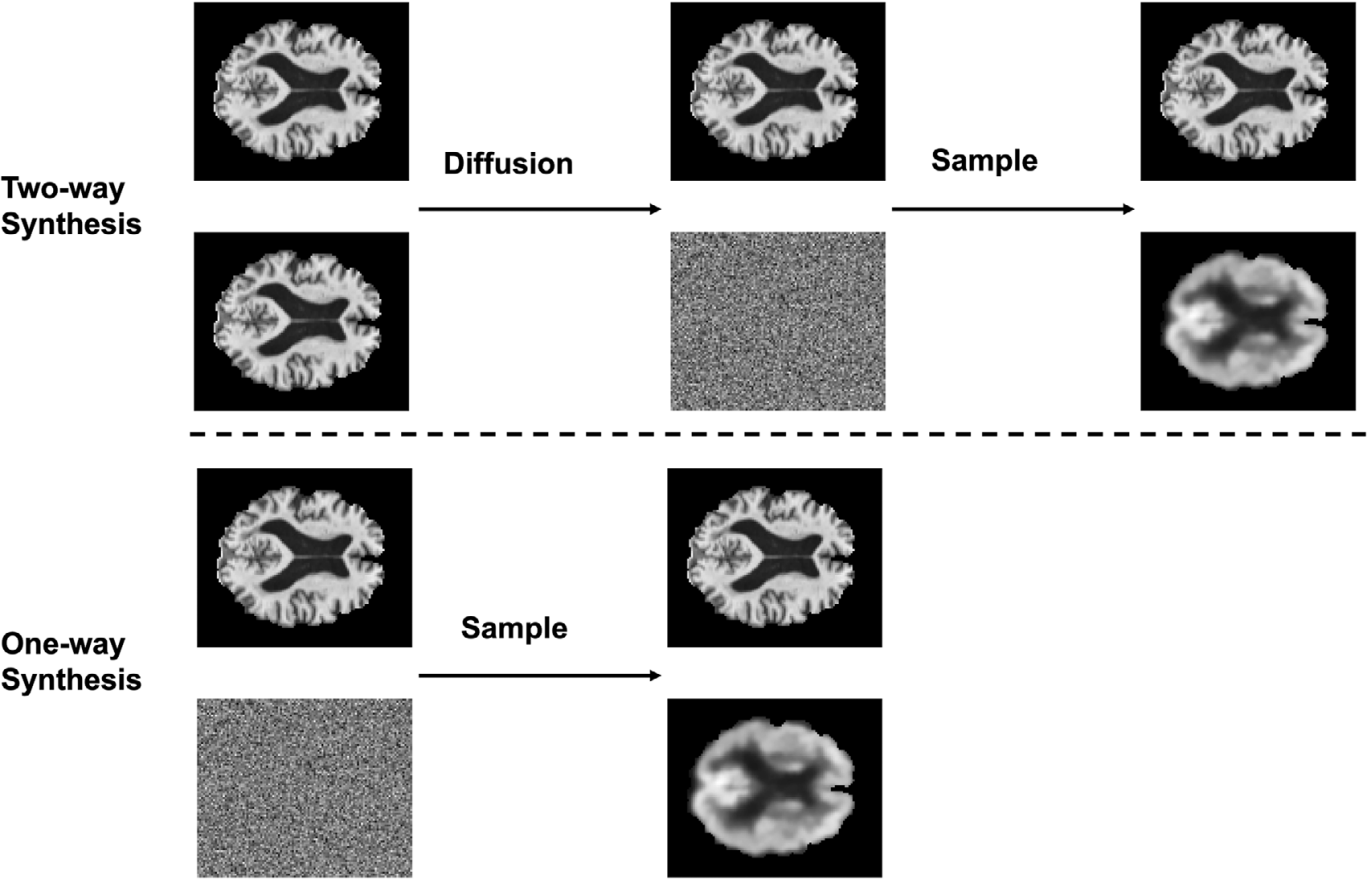
Illustration of the two approaches of synthesis, namely one‐way synthesis and two‐way synthesis.

Moreover, two approaches of synthesizing FDG PET from MRI T1WI using the above‐described diffusion model are proposed, namely one‐way synthesis and two‐way synthesis as shown in Figure 4. The one‐way synthesis approach starts with randomly generated Gaussian noise together with an MRI T1WI scan as the reference image. It only performs a sample process with an FDG PET–related class embedding, which tells the model to denoise the noised input to an FDG PET. The two‐way synthesis approach starts with an MRI T1WI scan together with the same MRI T1WI scan as reference image. It consists of two steps, namely diffusion and sample. In the diffusion process, an MRI T1WI–related class embedding tells the model to continuously add noise to the initial MRI T1WI to generate Gaussian‐distributed noise corresponding to the input, from which the input MRI T1WI can be sampled back. In the sample process, the model takes the noise derived from the diffusion process and continuously denoises the noise with an FDG PET–related class embedding to generate the synthesized FDG PET. The main difference between the one‐way and two‐way synthesis approaches is whether to introduce randomly generated noise for FDG PET synthesis. Due to no random noise introduced in the two‐way synthesis approach, the synthesized results would be deterministic while the results of the one‐way synthesis approach might change each time for model inferencing.

### 2.4 Classifier

To evaluate the effectiveness of the synthesized FDG PET scans in ML‐assisted AD diagnosis, we have designed a multilayer perceptron (MLP)–based classifier, which consists of several MLPs with each corresponding to one specific type of feature vector extracted from brain segments, namely WM, GM, and CSF, and returning the probability of each patient category (normal control [NC]/MCI/AD). The results from each MLP are then averaged to produce the final prediction. For a single modality, the final prediction is an average of three branches’ outputs with WM, GM, and CSF feature vectors accordingly. With multiple modalities, the prediction scores are further averaged to obtain a prediction score with multi‐modality data as joint input.

The input of the MLP‐based classifier is designed for the brain atlases‐based features extracted from different modalities with different brain segments, that is, GM, WM, and CSF, using AAL3^37^ atlases. As for MRI T1WI, the feature extraction happens by counting non‐zero voxels in each brain sub‐region defined in the selected brain atlases as MRI T1WI mostly reflects the degree of structural atrophy of the brain in AD diagnosis. However, for the FDG PET, feature extraction happens by summing the values in each brain sub‐region as the power reflects the degree of neuron activity, which is an important factor in the diagnosis of neurodegenerative diseases such as AD.

## 3 RESULTS

For the evaluation of FDG PET scans synthesized from MRI T1WI scans, five criteria metrics, namely mean absolute error (MAE), mean squared error (MSE), zero‐normalized cross correlation (ZNCC), structural similarity index measure (SSIM), and peak signal‐to‐noise rate (PSNR) scores, are used to evaluate the performance of the synthetic results. While computing the criteria metrics, the ground truth FDG PET scans and the synthesized images are both normalized to 0 to 1 to produce a standard comparison. The equations of criteria metrics are shown as follows.

(9) $$\begin{array}{c} MAE\;\left(X,Y\right)=\frac{\sum_{i=1}^{\xi }\left|y_{i}-x_{i}\right|}{\xi }\; \end{array}$$

(10) $$\begin{array}{c} MSE\;\left(X,Y\right)=\frac{\sum_{i=1}^{\xi }{\left(y_{i}-x_{i}\right)}^{2}}{\xi }\; \end{array}$$

(11) $$\begin{array}{c} NCC\left(X,Y\right)=\frac{1}{\xi }\;\frac{1}{\sigma_{X}\sigma_{Y}}\sum_{i=1}^{\xi }\left(x_{i}-\mu_{X}\right)\left(y_{i}-\mu_{Y}\right) \end{array}$$

(12) $$\begin{array}{c} SSIM\;\left(X,Y\right)=\frac{\left(2\mu_{X}\mu_{Y}+c_{1}\right)\left(2\sigma_{XY}+c_{2}\right)}{\left(\mu_{X}^{2}+\mu_{Y}^{2}+c_{1}\right)\left(\sigma_{X}^{2}+\sigma_{Y}^{2}+c_{2}\right)}\; \end{array}$$

(13) $$\begin{array}{c} PSNR\;\left(X,Y\right)=10log_{10}\left(\frac{MAX_{X,Y}^{2}}{MSE}\right) \end{array}$$

where $c_{1}={\left(k_{1}L\right)}^{2}$, $c_{2}={\left(k_{2}L\right)}^{2}$, and $c_{3}=c_{2}\;/2$ are used to stabilize the division with weak denominator and $k_{1}$ is set to 0.01, $k_{2}$ is set to 0.03. Meanwhile, *L* denotes the range of voxel values, μ denotes the mean value and σ denotes the standard deviation, and $\sigma_{XY}$ denotes the covariance of X,Y.

### 3.1 Experiments and results analysis for diffusion model

For the diffusion model, we have also conducted a five‐fold cross‐validation on the dataset. Moreover, instead of normalizing the image values to 0 ∼ 1, we have normalized the value of the input into the range –1 ∼ 1 with the formula:

(14) $$\mathrm{Normaliz}e_{-1∼1}\;\left(X\right)=\frac{X-\min\left(X\right)}{\max\left(X\right)-\min\left(X\right)}\;\times 2-1$$

As aforementioned in section 2.3, the class embedding determines the model's output of whether to generate MRI T1WI–related noises or FDG PET–related noises. Therefore, with the same trained model, we have designed two experiments for FDG PET synthesis. One experiment is the one‐way synthesis, which applies the sample process to the randomly generated noise data with MRI T1WI as the conditional image to obtain the desired output of synthesized FDG PET. The other experiment is the two‐way synthesis, which applies the diffusion process to the input MRI T1WI data first to generate the noise data and then applies the sample process to the generated noise data to obtain the desired output of synthesized FDG PET. An illustration of the two experiments is shown in Figure 4.

The experimental results are shown in Table 2 with two experiments denoted as “Diffusion Two‐way” and “Diffusion One‐way.” The two‐way experiment achieves an MAE score of 0.0187, an MSE score of 0.0024, a ZNCC score of 0.9823, an SSIM score of 0.9380, and a PSNR score of 26.47. The one‐way experiment has a slightly better performance than that of the two‐way experiment except for the SSIM score. It achieves an MAE score of 0.0274, an MSE score of 0.0044, a ZNCC score of 0.9814, an SSIM score of 0.9282, and a PSNR score of 23.83.

**TABLE 2:** Comparison of works with the same task as our proposed synthesis models for the prediction of FDG PET from MRI T1WI.

	MAE	MSE	ZNCC	SSIM	PSNR
Li et al.^29^	0.0862	–	–	0.5419	–
Sikka et al.^30^	0.0422	–	–	0.8211	–
Hu et al.^27^	–	–	–	0.89	**27.88**
Zhang et al.^28^	0.0318	–	–	0.7294	26.92
Vega et al.^32^	–	–	–	0.918	18.23
Vega et al.^31^	–	–	–	0.905	22.69
Ouyang et al.^33^	–	–	–	0.88	27.83
Diffusion one‐way	0.0274	0.0044	0.9814	0.9282	23.83
Diffusion two‐way	**0.0187**	**0.0024**	**0.9823**	**0.9380**	26.47

*Note*: For ZNCC, SSIM, and PSNR metrics, the higher value indicates the better performance for the prediction and for MAE and MSE metrics, the lower value indicates the better performance of the prediction. The metrics with the best performance are highlighted in bold.

Abbreviations: FDG PET, fluorodeoxyglucose positron emission tomography; MAE, mean absolute error; MRI T1WI, magnetic resonance imaging T1 weighted imaging; MSE, mean squared error; PSNR, peak signal‐to‐noise ratio; SSIM, structural similarity index measure; ZNCC, zero‐normalized cross correlation.

**FIGURE 5:**
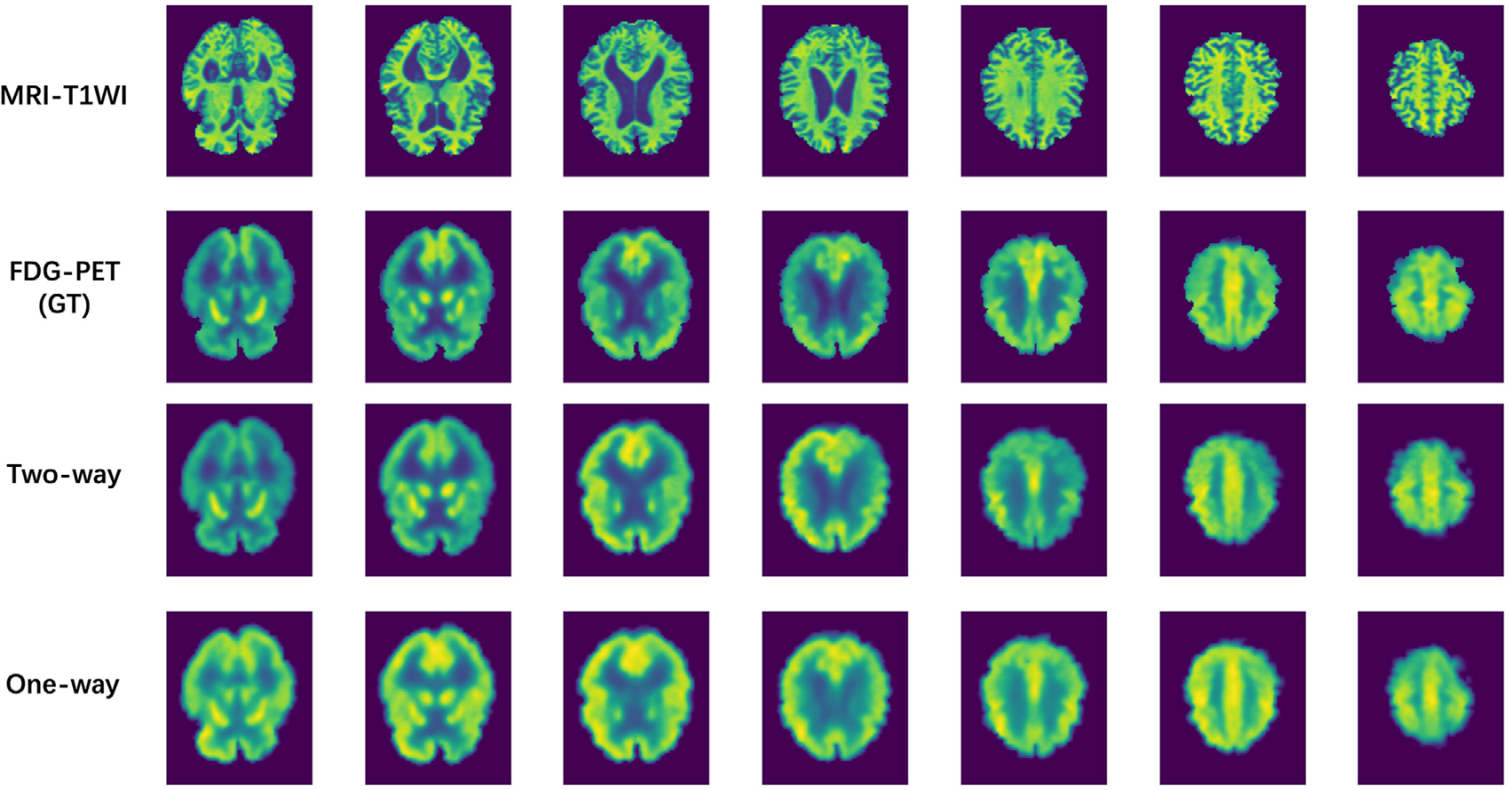
A visual comparison of the three synthetic results from a randomly selected sample. The first row is the conditional MRI T1WI, the second row is the ground truth FDG PET, the third and fourth rows are the synthetic results, two‐way diffusion and one‐way diffusion sequentially. FDG PET, fluorodeoxyglucose positron emission tomography; MRI T1WI, magnetic resonance imaging T1 weighted imaging.

A visual comparison of the three synthetic results from a randomly selected sample is shown in Figure 5

### 3.2 Experiments and results analysis for classifier

To evaluate the effectiveness of synthesis results, apart from comparing with the ground truth images using various criterion metrics, we have also designed and trained MLP‐based classifiers using different data modalities as input. Specifically, we experiment on a single modality including MRI T1WI, ground truth FDG PET, and synthesized FDG PET, and experiment on different combinations of joint input involving MRI T1WI.

The results of classifier performance for different types of input are shown in Table 3. For the classification of NC/MCI/AD, with MRI T1WI scans as input, the MLP classifier achieves an accuracy of 80.23%. With FDG PET as input, the MLP classifier achieves an accuracy of 81.31%, which outperforms the input setting using MRI T1WI only. With MRI T1WI and FDG PET as joint input, the MLP classifier also outperforms that of using MRI T1WI as input with an accuracy of 81.31%. For the two types of synthesized FDG PET scans as input, the MLP classifiers achieve accuracy of 79.16% and 81.41% for synthesized FDG‐PET scans derived from the one‐way diffusion model and two‐way diffusion model, respectively. The synthesized FDG PET scans derived from one‐way diffusion perform slightly worse than MRI T1WI but are still acceptable while that of two‐way diffusion performs better than MRI T1WI, which indicates that using the synthesized FDG PET scans for AD diagnosis is feasible. Moreover, combining synthesized FDG PET scan results with the MRI T1WI scans can produce a better overall performance compared to individual results as shown in Table 3. With MRI T1WI and synthesized FDG PET from one‐way diffusion, the MLP classifier achieves an accuracy of 81.90%, which even outperforms that of using MRI T1WI and ground truth FDG PET as input. Using MRI T1WI and synthesized FDG PET from two‐way diffusion as joint input achieves the best performance among our experiments with different input settings with an accuracy of 82.19%.

**TABLE 3:** Experimental results of MLP‐based classifiers with different types of input and their combinations.

NC/MCI/AD				
	Precision	Recall	F1 score	Accuracy
MRI T1WI				
NC	0.8893	0.8737	0.8814	80.23%
MCI	0.7874	0.8009	0.7941	
AD	0.7378	0.7296	0.7337	
FDG PET				
NC	0.8917	0.8667	0.8790	81.31% ↑
MCI	0.7910	0.8266	0.8084	
AD	0.7704	0.7333	0.7514	
One‐way diffusion				
NC	0.8737	0.8737	0.8737	79.16%
MCI	0.7732	0.8030	0.7878	
AD	0.7341	0.6852	0.7088	
Two‐way diffusion				
NC	0.8957	0.8737	0.8845	81.41% ↑
MCI	0.7926	0.8266	0.8092	
AD	0.7665	0.7296	0.7476	
MRI T1WI + FDG PET				
NC	0.8857	0.8702	0.8779	81.31% ↑
MCI	0.7918	0.8223	0.8067	
AD	0.7743	0.7370	0.7552	
MRI T1WI + one‐way				
NC	0.8979	0.8947	0.8963	81.90% ↑
MCI	0.8004	0.8158	0.8081	
AD	0.7672	0.7444	0.7556	
MRI T1WI + two‐way				
NC	0.8932	0.8807	0.8869	**82.19%** ↑
MCI	0.7967	0.8308	0.8134	
AD	0.7913	0.7444	0.7672	

NC/MCI				
	Precision	Recall	F1 score	Accuracy
MRI T1WI				
NC	0.8439	0.8912	0.8669	89.63%
MCI	0.9313	0.8994	0.9150	
FDG PET				
NC	0.8797	0.8982	0.8889	**91.49%** ↑
MCI	0.9371	0.9251	0.9310	
One‐way diffusion				
NC	0.8464	0.9088	0.8765	90.29% ↑
MCI	0.9417	0.8994	0.9200	
Two‐way diffusion				
NC	0.8741	0.9018	0.8877	91.36% ↑
MCI	0.9389	0.9208	0.9297	
MRI T1WI + FDG PET				
NC	0.8737	0.8982	0.8858	91.22% ↑
MCI	0.9368	0.9208	0.9287	
MRI T1WI + one‐way				
NC	0.8543	0.9053	0.8790	90.56% ↑
MCI	0.9400	0.9058	0.9226	
MRI T1WI + two‐way				
NC	0.8610	0.8912	0.8759	90.43% ↑
MCI	0.9322	0.9122	0.9221	

MCI/AD				
	Precision	Recall	F1 score	Accuracy
MRI T1WI				
MCI	0.8647	0.8758	0.8702	83.45%
AD	0.7803	0.7630	0.7715	
FDG PET				
MCI	0.8628	0.8887	0.8755	83.99% ↑
AD	0.7969	0.7556	0.7757	
One‐way diffusion				
MCI	0.8587	0.8587	0.8587	82.09%
AD	0.7556	0.7556	0.7556	
Two‐way diffusion				
MCI	0.8607	0.8865	0.8734	83.72% ↑
AD	0.7930	0.7519	0.7719	
MRI T1WI + FDG PET				
MCI	0.8695	0.8844	0.8769	**84.26%** ↑
AD	0.7939	0.7704	0.7820	
MRI T1WI + one‐way				
MCI	0.8650	0.8779	0.8714	83.58% ↑
AD	0.7833	0.7630	0.7730	
MRI T1WI + two‐way				
MCI	0.8628	0.8887	0.8755	83.99% ↑
AD	0.7969	0.7556	0.7757	

NC/AD				
	Precision	Recall	F1 score	Accuracy
MRI T1WI				
NC	0.9531	0.9263	0.9395	93.87%
AD	0.9245	0.9519	0.9380	
FDG PET				
NC	0.9313	0.9509	0.9410	93.87%
AD	0.9470	0.9259	0.9363	
One‐way diffusion				
NC	0.9437	0.9404	0.9420	**94.05%** ↑
AD	0.9373	0.9407	0.9390	
Two‐way diffusion				
NC	0.9496	0.9263	0.9378	93.69%
AD	0.9242	0.9481	0.9360	
MRI T1WI + FDG PET				
NC	0.9371	0.9404	0.9387	93.69%
AD	0.9368	0.9333	0.9351	
MRI T1WI + one‐way				
NC	0.9399	0.9333	0.9366	93.51%
AD	0.9301	0.9370	0.9336	
MRI T1WI + two‐way				
NC	0.9532	0.9298	0.9414	**94.05%** ↑
AD	0.9278	0.9519	0.9397	

*Note*: The best accuracies of experiments with different tasks are highlighted in bold.

Abbreviations: AD, Alzheimer's disease; FDG PET, fluorodeoxyglucose positron emission tomography; MAE, mean absolute error; MCI, mild cognitive impairment; MLP, multilayer perceptron; MRI T1WI, magnetic resonance imaging T1 weighted; MSE, mean squared error; NC, normal control; PSNR, peak signal‐to‐noise ratio; SSIM, structural similarity index measure; ZNCC, zero‐normalized cross correlation.

We have also conducted experiments on binary classification, namely NC/MCI, MCI/AD, and NC/AD, in which the settings for each binary classification are the same as the three‐class classification of NC/MCI/AD. The results are also shown in Table 3, in which using FDG PET as input achieves the best performance for NC/MCI with an accuracy of 91.49%, using MRI T1WI and FDG PET as joint input achieves the best performance for MCI/AD with an accuracy of 84.26%, and using MRI T1WI and synthesized FDG PET scans derived from the two‐way diffusion model as joint input achieves the best performance for NC/AD with an accuracy of 94.05%. Though using MRI T1WI and synthesized FDG PET scans derived from the two‐way diffusion model as joint input only achieves the best performance on NC/AD classification, it still indicates that involving synthesized FDG PET scans derived from the two‐way diffusion model can enhance the classification performance compared to using MRI T1WI as input. Meanwhile, using synthesized FDG PET scans derived from the one‐way diffusion model can also enhance the classification performance for NC/MCI and MCI/AD, except for NC/AD. In conclusion, the results show the feasibility of the synthesized model in computer‐aided diagnosis for AD.

## 4 DISCUSSION

From the view of FDG PET synthesis and according to Table 2 and Figure 5, two‐way synthesis outperforms one‐way synthesis by using diffusion models both from the visual check and criterion metrics. As described by Xiao et al.,^38^ the diffusion models have the advantages of both “high‐quality samples” and “model converge/diversity.” The reason the two‐way synthesis outperforms the one‐way synthesis might be that the one‐way synthesis introduces randomly generated noise in the synthesis process, which increases the bias and thus reduces the quality of the synthesized FDG PET views. Though the one‐way synthesis performs worse than the two‐way synthesis, it still looks similar to the ground truth FDG PET scans.

As using both the one‐way synthesis and two‐way synthesis for FDG PET views can produce convincing results for AD diagnosis, thus they are both effective in the clinic. Meanwhile, the complementary views of synthesized FDG PET scans with MRI T1WI scans can enhance the overall performance of ML‐based AD diagnosis, which implies the synthesized FDG PET scans not only look similar to the ground truth FDG PET scans but also make contributions to AD diagnosis. Furthermore, comparing the performance of using MRI T1WI to synthesized FDG PET scans derived from either the one‐way or two‐way diffusion model, it appears that the two‐way synthesis produces better results compared to the one‐way synthesis for NC/MCI/AD, MCI/AD, and NC/AD while opposite for NC/MCI. A similar situation exists for NC/AD when using synthesized scans as input only. Considering the overall classification performance together with the criterion metrics for image similarity measure, we still hypothesize that two‐way synthesis generally outperforms one‐way synthesis. However, according to the results, one‐way synthesis is still valuable for enhancing the performance of ML‐based AD diagnosis. Biases introduced by one‐way synthesis might also help in the training process to overcome the overfitting problem, especially for the limited‐scale dataset.

Our experimental results demonstrate that the involvement of an additional view, specifically FDG PET in this work, can enhance the performance of ML‐based models for AD diagnosis, even though the involved view is synthesized instead of ground truth. Therefore, we consider the generality that more views, including but not limited to image data, can also enhance the model's performance. As many disease‐related medical data are hard or costly to acquire, for future works, involving more views, either of real data or of synthesized data, this would be a feasible way to improve the performance of ML‐based models for AD diagnosis.

## 5 CONCLUSION

This paper proposes a diffusion model with two strategies to synthesize FDG PET scans from MRI T1WI scans for AD diagnosis. In our proposal, the two‐way synthesis achieves state‐of‐the‐art results with MAE at 0.0187, MSE at 0.0024, ZNCC at 0.9823, SSIM at 0.9380, and PSNR at 26.47. The one‐way synthesis also shows convincing results with SSIM scores of 0.9282.

Further to the evaluation of the synthesized FDG PET scans compared to the ground truth FDG PET scans, we have also designed MLP‐based classifiers to carry out validation. The experiments show positive results and prove the feasibility of synthesized FDG PET scans in AD diagnosis and the ability to improve the classifiers’ performance.

This work gives support to the notion that ML‐based cross‐domain data synthesis can be a useful approach to improve the diagnosis of AD by providing additional synthesized disease‐related views for multi‐view learning.

## CONFLICT OF INTEREST STATEMENT

The authors declare that they have no known competing financial interests or personal relationships that could have appeared to influence the work reported in this paper. Author disclosures are available in the supporting information.

## Supporting information



Supporting Information
